# Proof of principle: quality control of therapeutic cell preparations using senescence-associated DNA-methylation changes

**DOI:** 10.1186/1756-0500-7-254

**Published:** 2014-04-23

**Authors:** Anne Schellenberg, Sébastien Mauen, Carmen Mareike Koch, Ralph Jans, Peter de Waele, Wolfgang Wagner

**Affiliations:** 1 Helmholtz-Institute for Biomedical Technology, Stem Cell Biology and Cellular Engineering, RWTH Aachen University Medical School, Pauwelsstrasse 20, 52074 Aachen, Germany; 2 Cardio3 Biosciences, Rue Edouard Belin 12, 1435 Mont-Saint-Guilbert, Belgium

**Keywords:** Mesenchymal stem cells, Long-term culture, Expansion, Senescence, Epigenetic, DNA-methylation, GMP, Quality control

## Abstract

**Background:**

Tracking of replicative senescence is of fundamental relevance in cellular therapy. Cell preparations – such as mesenchymal stromal cells (MSCs) - undergo continuous changes during culture expansion, which is reflected by impaired proliferation and loss of differentiation potential. This process is associated with epigenetic modifications: during *in vitro* culture, cells acquire senescence-associated DNA methylation (SA-DNAm) changes at specific sites in the genome. We have recently described an Epigenetic-Senescence-Signature that facilitates prediction of the state of cellular aging by analysis of DNAm at six CpG sites (associated with the genes *GRM7*, *CASR*, *PRAMEF2*, *SELP*, *CASP14* and *KRTAP13-3*), but this has not yet been proven over subsequent passages and with MSCs isolated under good manufacturing practice (GMP) conditions.

**Findings:**

MSCs were isolated from human bone marrow and GMP-conform expanded for up to 11 passages. Cumulative population doublings (cPDs) and long-term growth curves were calculated based on cell numbers at each passage. Furthermore, 32 cryopreserved aliquots of these cell preparations were retrospectively analyzed using our Epigenetic-Senescence-Signature: DNAm-level was analyzed at six specific CpGs, and the results were used to estimate cPDs, time of culture expansion, and passage numbers. Overall, predicted and real parameters revealed a good correlation, particularly in cPDs. Based on predicted cPDs we could reconstruct long-term growth curves and demonstrated the continuous increase in replicative senescence on molecular level.

**Conclusion:**

Epigenetic analysis of specific CpG sites in the genome can be used to estimate the state of cellular aging for quality control of therapeutic cell products.

## Findings

Mesenchymal stromal cells are usually culture expanded for clinical application. Yet, cells undergo tremendous changes during long-term culture: they enlarge, acquire flat “fried-egg” morphology, lose differentiation potential, and ultimately stop proliferation [1,2]. This phenomenon has already been described over 50 years ago by Leonhard Hayflick [3]. It is important to note that functional changes - including loss of colony-forming units, as well as adipogenic and osteogenic differentiation potential - become already evident during early passages [4]. There is also evidence that secretory and immunomodulatory functions of MSCs change upon *in vitro* expansion [5]. Furthermore, cells in culture may be more prone to mutations – and hence, malignant transformation [6,7]. This conflicts with the high demands for standardization and safety in regenerative medicine. Therefore, it is important to monitor replicative senescence in long-term culture of MSCs [8].

Analysis of senescence-associated beta-galactosidase (SA-β-Gal) activity is the most widely used biomarker for replicative senescence *in vitro*[9]. We observed that it is particularly detected in very late, senescent passages [10], although other authors indicated that it is also up-regulated earlier in culture [11,12]. Furthermore, SA-β-Gal may also be induced upon stress during *in vitro* culture [13] but this analysis does not facilitate reliable estimation of cumulative population doublings (cPDs). Telomere attrition has also been correlated with replicative potential, but the results vary between different cell types and culture methods [14-16]. Analysis of cPDs necessitates precise cell counting at each passage throughout culture expansion. So far, the state of cellular aging could not be determined retrospectively without this information.

**Figure 1:**
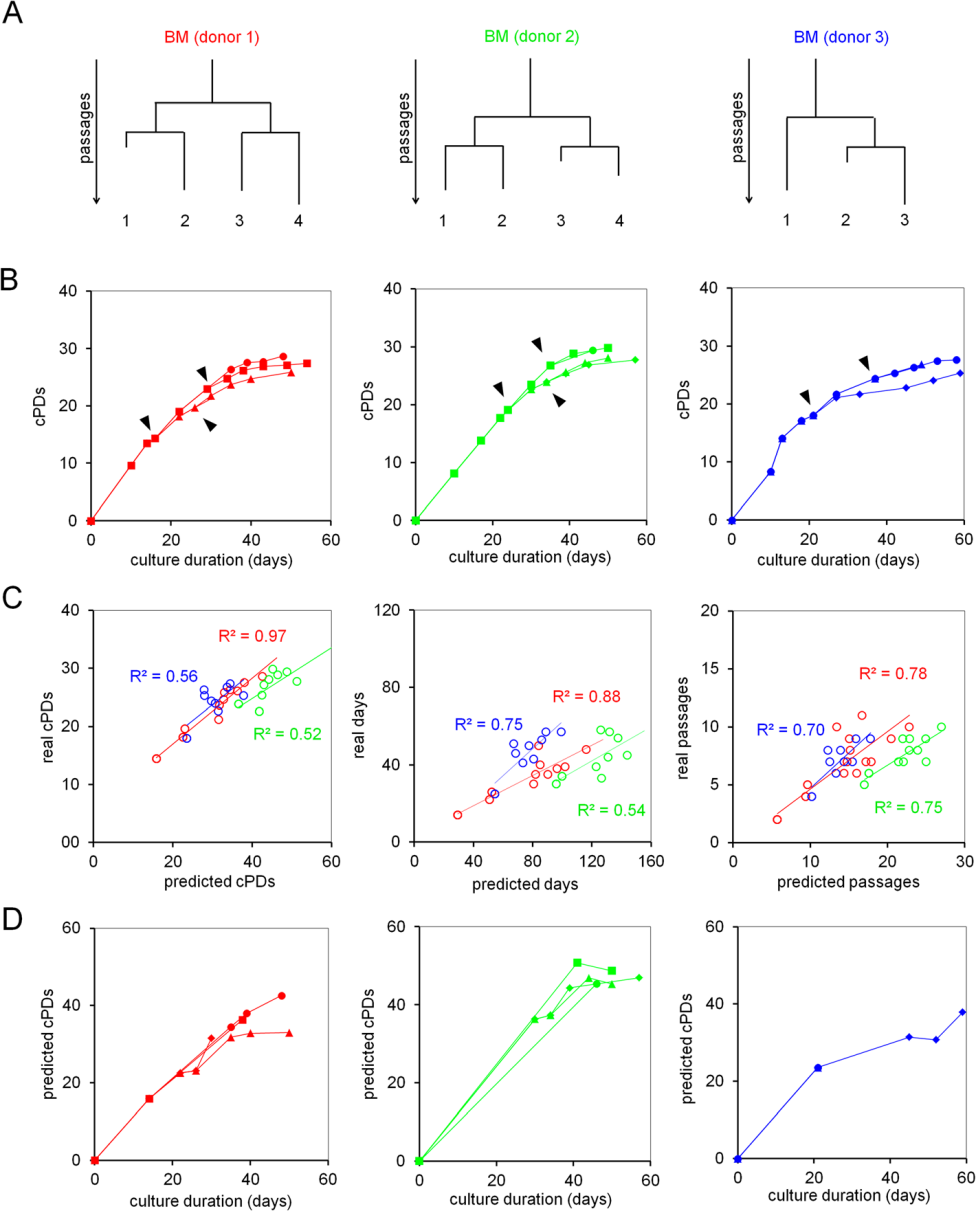
**Epigenetic-Senescence-Signature during long-term culture of MSCs. (A)** MSCs were isolated form bone marrow (BM) of three patients and sub-cultured during expansion as indicated by the hierarchical trees. **(B)** Long-term growth curves reveal that cell growth decayed within 30 cumulative population doublings (cPDs). Black triangles indicate subculturing as mentioned above. **(C)** DNAm analysis at six senescence-associated CpG sites was then performed in various cryopreserved vials by pyrosequencing [22,24]. Based on these results we calculated predictions for cPDs, days of *in vitro* culture and passage numbers. These predictions were subsequently compared to real values. **(D)** Predictions for cPDs, as determined using the Epigenetic-Aging-Signature, were plotted against real culture time to simulate long-term growth curves. Overall, the results correspond to real long-term growth curves even with regard to different subcultures (symbols as depicted in B).

Culture expansion of MSCs is associated with senescence-associated DNA-methylation (SA-DNAm) changes at specific sites in the genome which become either hyper-methylated or hypo-methylated [17,18]. These senescence-associated CpG sites are enriched in developmental genes and they correlate with repressive histone marks [19]. The mechanism regulating these SA-DNAm changes is yet unclear – it is possible that they resemble a kind of epigenetic drift, similar to observations in aging of the organism [20]. On the other hand, we have recently demonstrated that almost the entire set of SA-DNAm is reversed by reprogramming into induced pluripotent stem cells (iPSCs) indicating that the process can be reversed by the pluripotent state [21]. It is also unknown if SA-DNAm changes entail the profound functional changes during culture expansion, or if they rather resemble a byproduct. Either way, SA-DNAm changes are highly reproducible and may therefore be used to monitor cellular senescence. To this end, we have elaborated an Epigenetic-Aging-Signature based on six specific CpG sites which seemed to display consistent SA-DNAm changes in different cell preparations. Integration of these DNAm levels in linear-regression models facilitated prediction of passage number, cPDs, and days of *in vitro* culture [22]. Yet, this method required further validation – particularly on cell preparations isolated under good manufacturing practice (GMP) conditions. So far, the method has not been used with cells isolated in serial passages and with DNA directly isolated from cryopreserved cell aliquots. Therefore, we performed the following retrospective study:

MSCs were isolated from human bone marrow of the iliac crest of three different heart patient donors after full informed consent with ethical approval by the Ethical Committee from the University Hospital Erasme of the Université Libre de Bruxelles (ULB; aggregation number N°OM021) and cultivated as described before [23]. In brief, cells were cultured at 37°C in Advanced Minimal Essential Medium (Invitrogen, Eugene, OR, USA) supplemented with 5% human platelet lysate (Mill Creek Life Science, Rochester, MN, USA), Glutamax™ (Invitrogen), and penicillin/streptomycin (Invitrogen). After 24 h, non-adherent bone marrow and cellular debris were removed and adherent mesenchymal cells were expanded using cell seeding densities varying between 2,000 and 6,000 cells/cm^2^. After the initial passages cultures were split into parallel subcultures, cultivated for several passages and in some cases subcultured for a second time (Figure 1A). Upon several amplification rounds cells were treated with C3BS proprietary cardiotrophic cocktail containing additional growth factors. Post-cocktail treatment, cells were cultured for a minimum of two additional passages or until growth arrest was observed. At each passage, cell numbers and seeding density have been carefully documented. Based on these numbers we calculated long-term growth curves (Figure 1B). MSCs of all three donors stopped proliferation after about 30 cPDs. Even when the culture was split and further cultured independently, there was only a slight deviation in the maximal number of cPDs which might either be due to the outgrowth of different subfractions or deviations in cell counting.

Thirty-two cryopreserved samples, which correspond to various passages of the three donors, were then analyzed using the Epigenetic-Senescence-Signature to estimate the state of cellular aging. In our previous work, we did not analyze many subsequent passages from the same cell preparation and harvested DNA directly from cells in culture. DNA was now isolated from cryopreserved vials without taking cells into culture and then bisulfite-converted. SA-DNAm of the six relevant CpGs was analyzed in a blinded manner by pyrosequencing as described in detail before (performed at Varionostic GmbH, Ulm, Germany) [22,24]. In brief, two CpGs become continuously hyper-methylated in long-term culture (associated with the genes *GRM7* and *CASR*) and four CpGs become hypo-methylated (corresponding to *PRAMEF2*, *SELP*, *CASP14* and *KRTAP13-3*). DNAm values of each sample were then used for linear regression analysis to predict cPDs, passage number and days of *in vitro* culture. An online-calculator is provided under: http://www.molcell.rwth-aachen.de/dms/. It needs to be noted that the genes associated with the six relevant CpGs are not expressed in MSCs [22]. In fact, there are highly reproducible changes in gene expression profiles during long-term culture [10,25] but this seems to be hardly related to SA-DNAm changes [17,21]. Thus, there is no direct association of the Epigenetic-Senescence-Signature with well-known pathways of senescence [26] – it is a suitable biomarker but the functional relevance is yet unclear.

Overall, the predictions for cPDs, days of *in vitro* culture and passage numbers correlated well with real parameters, although they were slightly overestimated (Figure 1C). The best correlation was observed with cPDs which may be not surprising – passage numbers resemble a rather imprecise measure which is greatly affected by handling in cell culture, whereas cultivation-time is interdependent on donor-specific differences in cell growth. Overestimation of the state of cellular aging might be attributed to operational procedures in cell culture or to systematic deviation in cell counting. On the other hand, differences in starting material and culture conditions may have significant impact on replicative senescence during culture expansion, too, and this may necessitate further adaptation of the differential equations. Particularly samples of donor 3 revealed a low correlation and a different slope of predicted *versus* real parameters. This supports the notion that there are differences in the state of cellular senescence between cell preparations which are not necessarily reflected by long-term growth curves. Subsequently, we simulated long-term growth curves based on the predicted values for cPDs. As mentioned above, different subcultures reached slightly different maximal cPDs. The same tendencies were also observed epigenetically: subcultures which reached more real cPDs were also estimated to have reached more cPDs using the Epigenetic-Aging-Signature (Figure 1D). This indicates that the differences are indeed cell-intrinsic and not due to deviations in cell counting.

There is a growing perception that standardized protocols and quality control of therapeutic cell preparations are a prerequisite for reliable and reproducible cellular therapy. Given the major impact of long-term culture on molecular changes in MSCs it is very likely that this process has also impact on the therapeutic potential. Clinical outcome should therefore be evaluated in the context of cellular aging. In this short report, we provide further evidence that this can be tracked by specific epigenetic modifications. Epigenetic analysis may be used as a tool to validate cell counts and sampling in the course of long-term culture of MSCs – which is a time consuming and error prone procedure – and it is of particular relevance if detailed cell numbers have not been documented throughout expansion. We demonstrate that our Epigenetic-Senescence-Signature reflects inter-individual differences and variation in subpopulations which are not necessarily reflected in conventional long-term growth curves. In this regard, the epigenetic state of cell preparations might even provide the more accurate measurement for the biological state of cellular aging. Furthermore, it will be interesting to investigate if molecules secreted by senescent cells, a phenomenon termed senescence-associated secretory phenotype (SASP), affect also the epigenetic state of cells in culture [27,28]. Our method facilitates analysis of continuously cultured cells as well as retrospective analysis of cryopreserved residual vials [22]. Cryopreservation is commonly performed in many labs and clinical trials – even though this process makes a selection because, after thawing, not all cells grow out. On the other hand, applicability on such retained samples, which are usually collected in clinical trials, now opens new perceptive to gain better insight in the impact of long-term culture on clinical outcome. In the future, it would therefore be interesting to analyze SA-DNAm samples in therapeutic cellular products and to correlate the results with clinical performance.

## Abbreviations

MSCs: Mesenchymal stromal cells; SA-β-Gal: Senescence-associated beta-galactosidase; cPDs: cumulative population doublings; DNAm: DNA methylation; SA-DNAm: Senescence-associated DNA-methylation; iPSCs: induced pluripotent stem cells; GMP: Good manufacturing practice; BM: Bone marrow.

## Competing interests

RWTH Aachen has applied for a patent application for the “Epigenetic-Senescence-Signature”. WW is involved in the company Cygenia that provides this service to other researchers. SM & PdW are employed by Cardio3 Biosciences; RJ has been employed by Cardio3 Biosciences. Apart from this, the authors have no competing interest for this study.

## Authors’ contributions

AS analyzed the data and revised the manuscript. SM collected the data, participated in the analysis of the data and the writing of the manuscript. CMK collected and analyzed the data. RJ participated in the design of the study. PW participated in the design of the study and the writing of the manuscript. WW carried out the design of the study and the writing of the manuscript. All authors read and approved the final manuscript.
